# PHYSICAL FRAILTY IN OLDER ADULTS: WHAT DRIVES AND PREVENTS IT?

**DOI:** 10.1093/geroni/igae098.1117

**Published:** 2024-12-31

**Authors:** Jeremy Walston

**Affiliations:** Johns Hopkins University, Baltimore, Maryland, United States

## Abstract

Physical frailty puts older adults at very high risk of adverse health outcomes and early mortality. There are multiple physiological and molecular changes that drive the development of frailty, including altered metabolism, activated inflammatory pathways, and autonomic nervous system dysregulation. Over the past several decades, our team has worked to identify specific physiological and molecular changes that drive earlier onset of frailty and late life decline in older adults. These pathway discoveries have also informed the underpinnings of physical resiliency in a subset of older adults, and helped to identify potential mechanisms by which older adults can live at lower biological risk of developing illnesses as well as functional and cognitive decline. This lecture will encapsulate how the evolving understanding of physical frailty, underpinned by decades of research, is reshaping our approach to healthy aging and reflecting a profound shift in the landscape of gerontology research.

